# The Patient's Gender Influencing the Accuracy of Diagnosis and Proposed Sepsis Treatment in Constructed Cases

**DOI:** 10.1155/2020/4823095

**Published:** 2020-07-21

**Authors:** Andreas Pikwer, Madeleine Carlsson, Duraid Abod Mahmoud, Markus Castegren

**Affiliations:** ^1^Centre for Clinical Research Sörmland, Uppsala University, Uppsala, Sweden; ^2^Department of Clinical Sciences at Malmö, Anaesthesiology and Intensive Care, Lund University, Lund, Sweden; ^3^Perioperative Medicine and Intensive Care (PMI), Karolinska University Hospital and CLINTEC and FyFa, Karolinska Institutet, Stockholm, Sweden; ^4^Department of Medical Sciences, Infectious Diseases, Uppsala University, Uppsala, Sweden

## Abstract

**Background:**

Male sex is an independent risk factor for sepsis development. In addition to immunological gender differences, women less often receive sepsis treatment once diagnosed. Gender differences have also been described in other medical conditions, such as acute coronary syndrome.

**Aim:**

To study whether the gender of patients influenced physicians' tendency to suspect sepsis and propose correct initial sepsis treatment in constructed cases.

**Method:**

Four cases were constructed to fulfil the sepsis-3 criteria as well as raise clinical suspicions of other common medical differential diagnoses. Two of the cases were drafted in two versions, only differing in the gender of the patient. The two versions were randomly distributed to all clinical physicians in a medical region in Sweden. The responding physicians were asked to state the three most important diagnoses and the three most important initial treatments for each case. If sepsis were among the stated diagnoses together with fluids and antibiotics, the case was considered as correctly identified and initially treated sepsis.

**Results:**

120 hospital physicians answered the cases. In the case the patient was a female, the respondents correctly identified and treated sepsis significantly more often than if the patient was of the male sex (Case 1: 12/58 vs 2/62, *p* < 0.01 and Case 2: 25/62 vs 13/58, *p* < 0.05).

**Conclusion:**

A low proportion of Swedish physicians identified and proposed treatment for sepsis in four constructed cases. In the case the patient strongly mimicked other diagnoses common in the male sex, the male cases were less often correctly identified and treated for sepsis.

## 1. Introduction

Sepsis is one of the leading causes of death in the intensive care unit (ICU), with mortality in severe sepsis and septic shock approximating 20% [1–3]. Sepsis is currently defined as life-threatening organ dysfunction caused by a dysregulated host response to infection [4]. In 2016, the definitions of sepsis and septic shock underwent a revision, known as the sepsis-3 consensus definitions [5]. In the surviving sepsis campaign guidelines, strong recommendations are given to start intravenous antimicrobial treatment within the first hour and fluid resuscitation within the first three hours when a patient fulfills the criteria for sepsis, and in the latter case, when signs of sepsis-induced hypoperfusion are present [6].

Several studies have identified male sex as an independent risk factor for the development of severe infection and sepsis [7–9]. This can in part be attributed to the effects of sex hormones, where androgens have been shown to have immunosuppressive effects in contrast to female sex hormones [9–12]. In the large epidemiological study by Martin et al., men had a higher relative risk (1.28) than women to have sepsis [1] even though the incidence of sepsis among women increased more rapidly than among men over the twenty-year period the study covered.

In addition to immunological differences between men and women, it has been shown that sex could influence the management of septic patients. Mikkelsen et al. showed that female sex was associated with a lower usage of early goal-directed therapy [13] in the emergency department. An investigation showed that women with severe sepsis or septic shock were less likely than men to receive antibiotics within three hours from diagnosis [14].

There are sex-associated differences in the management and outcome in several medical emergencies, such as acute coronary syndrome, opioid withdrawal, liver failure, and diabetic ketoacidosis [15–19]. These conditions could, because of resemblance in the clinical presentation, be misinterpreted as sepsis or vice versa.

To further discern the reasons for gender disparities, we aimed to investigate whether the sex of the patient affects the clinicians' decision-making when prioritizing between differential diagnoses. To study this, we used a questionnaire-based case study design with the possibility to randomize the sex of the patient with all other variables unchanged. The design allowed the constructed cases to fulfil diagnostic criteria for sepsis as well as strongly resemble other medical conditions. The aim of this investigation was to study whether the sex of the patient influenced the clinical practitioners' ability to diagnose sepsis and order subsequent correct initial treatment, such as fluid resuscitation and antibiotics.

## 2. Materials and Methods

**Figure 1:**
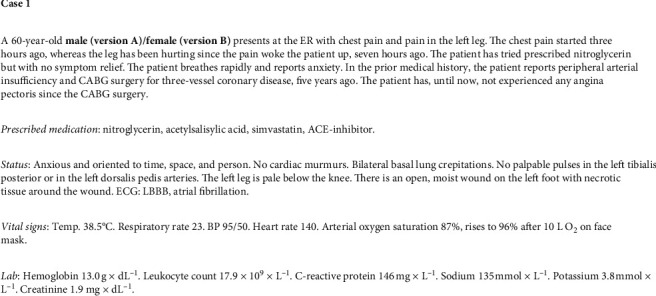
Case 1.

**Figure 2:**
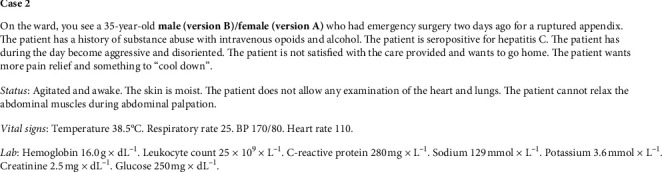
Case 2.

**Figure 3:**
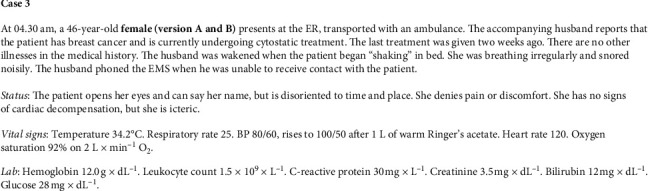
Case 3.

**Figure 4:**
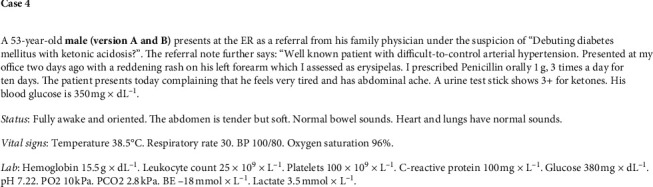
Case 4.

263 physicians with varying experience and from different specialties were asked to anonymously answer a questionnaire containing questions on four constructed patient cases, Figures 1–4. The questionnaires were sent by e-mail to all physicians working at the County Hospital, Mälarsjukhuset, in the north-western area of the County of Sörmland. The county is in the Mälardalen area of central Sweden. The county's largest hospital is providing specialized health care for 130000 inhabitants as well as being a referral hospital for 140000 additional inhabitants. In the e-mail, the physicians were asked to voluntarily answer the questions and to return the questionnaires by regular mail, to maintain anonymity. The physicians were informed that the purpose of the study was to assess the general level of knowledge of initial treatment to patients with emergency symptoms of critical disease. One month after the initial e-mail, all physicians received a reminding e-mail, asking the ones that had yet not participated in the study to answer the questionnaires.

All four patient cases were constructed to fulfil the diagnostic criteria for severe sepsis according to the international sepsis definitions conference of 2001, so called the sepsis-2 criteria, i.e., >2 criteria of systemic inflammatory response syndrome (SIRS) as well as at least one organ dysfunction [20]. All cases also fulfilled the diagnostic criteria for sepsis according to the sepsis-3 criteria [5]. All cases were constructed to show signs of probable sepsis-induced hypoperfusion (hypotension, tachycardia, or hyperlactatemia) where, according to the international guidelines for sepsis and septic shock [6], fluid treatment and early empiric antibiotic treatment are indicated. The cases were also constructed to resemble four different differential diagnoses, i.e., acute coronary syndrome, opioid withdrawal, liver failure, and diabetic ketoacidosis. The rationale for this was that a number of missed sepsis diagnoses in several different patients were discussed in morbidity and mortality rounds at the ICU, where patients had been primarily treated for other conditions than sepsis but were later rediagnosed as sepsis due to positive growth in blood cultures. The four aforementioned conditions were identified as differential diagnoses where sepsis easily could be missed. The four cases were nonauthentic, but inspired by patients that had primarily been diagnosed for other conditions than sepsis. The authors of this article are clinical experts and researchers in emergency medicine and intensive care medicine and jointly constructed the four cases. The cases were constructed in two versions, A and B. The versions differed in only one aspect, i.e., the sex of the patients in patient Cases 1 and 2. In Cases 3 and 4, the sex of the patients was the same in both versions. In version A of the questionnaire, the patient in Case 1 was a male, whereas the patient in Case 2 was a female. In version B, the patients in Cases 1 and 2, were female and male, respectively. In both versions, the patient in Case 3 was female and male in Case 4. Which version of the questionnaire that has to be sent out to each participant was randomly chosen. All cases can be seen in Figures 1–4.

In each case, the participants were asked to identify the three most probable and important-to-treat differential diagnoses and propose the three most important primary measures to be carried out and to assess at which level of care the patient should initially be treated, i.e., either at a general ward, at a high-dependence ward, or at an intensive care unit. In addition to answering the questions above, the participants were asked to give their corresponding age span (20–30, 30–40, 50–60, or 60+ years), their sex, and their position (intern, resident, consultant, or senior consultant), as well as their field of medical specialty.

### 2.1. Ethics

According to the Swedish legislation, the study did not need ethical clearance by the Swedish ethical review authority since all cases were constructed and nonauthentic and all participants were asked to answer the cases anonymously.

### 2.2. Treatment of Data and Statistics

All answers were transferred to a data sheet after the termination of the study. A variable named “correct identification and initial sepsis treatment” was constructed in the data sheet. If a participant had identified sepsis as one of the three most important and probable diagnoses as well as proposed antibiotics and fluid administration to be among the three most important measures to be carried out in a case, that case was given the value 1 on “correct identification and initial sepsis treatment.” Failure to either identify sepsis or propose antibiotics or fluid treatment yielded the value 0.

The frequencies of correct identification and initial sepsis treatment, sepsis among the three most important diagnoses, and proposed fluid and antibiotics among the three most important measures to be carried out, as well as ICU as the appropriate level of care, were compared between the questionnaire versions A and B and analysed for differences with Chi^2^ tests and Fisher's exact tests, where appropriate. A *p* value of <0.05 was considered statistically significant. Statistica (Statsoft, Tulsa, OK, USA) was used in the statistical calculations.

## 3. Results

In total, 120 participants answered a questionnaire and returned them anonymously by regular mail, rendering a response frequency of 45.7%. The participants were evenly distributed among the age groups, position, and medical specialties, Table 1. When the participants that had completed versions A and B were compared, no significant differences concerning the age span, position, or medical specialty were seen, Table 1.

### 3.1. Case 1

The most common diagnoses proposed by the participants in both A and B versions were myocardial ischemia, followed by pulmonary embolism and acute arterial thromboembolism. These three diagnoses were proposed by 93, 77, and 69% of all participants, respectively, as being among the three most important and probable differential diagnoses. Only 36/120 of the participants had proposed sepsis as one of the three most important diagnoses.

A significantly higher part of the participants that had answered version B of the questionnaire, i.e., where the patient was of female sex, had correctly identified sepsis as an important differential diagnosis as well as proposed both antibiotics and fluids among the three most important initial therapeutic measures, Table 2. In the answers of version B, the participants had proposed that antibiotics and fluids belonged to the three most important initial measures to a higher degree than in version A, Table 2. Approximately, 60% proposed ICU as the appropriate level of care with no differences between versions A and B of the questionnaire.

### 3.2. Case 2

The most common diagnoses proposed by the participants were sepsis followed by drug withdrawal symptoms and postoperative complications (i.e., postoperative bleeding, postoperative infection, bowel perforation, and ileus). These three diagnoses were proposed by 88, 73, and 42% of all participants, respectively, as being among the three most important and probable differential diagnoses. There were no differences when the participants' answers regarding diagnosis between versions A and B were compared.

Compared to the participants that had answered version B, a significantly higher part of the participants that had answered version A of the questionnaire, i.e., where the patient was of the female sex, had correctly identified sepsis as an important differential diagnosis as well as proposed both antibiotics and fluids among the three most important initial therapeutic measures, Table 2. In the answers of version A, the participants had proposed that fluids belonged to the three most important initial measures to a higher degree than in version B, Table 2. Approximately, 52% had proposed ICU as the appropriate level of care with no differences between versions A and B of the questionnaire.

### 3.3. Control Cases, 3 and 4

In Case 3, the most common diagnosis proposed by the participants was sepsis, followed by brain metastases associated with epilepsy and liver failure. These three diagnoses were proposed by 85, 40, and 39% of all participants, respectively, as being among the three most important and probable differential diagnoses. Approximately, 77% had proposed ICU as the appropriate level of care. In total, 39% of the physicians proposed sepsis as an important differential diagnosis and proposed antibiotics and fluid resuscitation among the most important initial therapeutic measures, with no differences between the participants that answered the version A or B of the questionnaire.

In Case 4, the most common diagnoses proposed by the participants were diabetic ketoacidosis, sepsis, and infection. These three diagnoses were proposed by 100, 72, and 25% of all participants, respectively, as being among the three most important and probable differential diagnoses. Approximately, 59% had proposed ICU as the appropriate level of care. In total, 55% of the physicians proposed sepsis as an important differential diagnosis and proposed antibiotics and fluid resuscitation among the most important initial therapeutic measures, with no differences between the participants that answered the version A or B of the questionnaire.

## 4. Discussion

The current study found that, keeping all other factors the same, just changing the sex of the patients in constructed cases affected the physicians' ability to correctly identify sepsis and suggest correct initial sepsis treatment. In the case of a female patient, a correct sepsis diagnosis and initial treatment was more likely.

The reason for this difference is not obvious and contradicts previous clinical observations [1, 13, 14]. In this study, all the cases showed symptoms of sepsis, but the scenarios also strongly mimicked other medical emergencies. A possible explanation is that the physicians' estimation of the probability of the differential diagnoses such as acute coronary syndrome and opioid withdrawal depended on the sex of the patient, and swayed the physicians to focus their initial treatment suggestions on those diagnoses.

The symptoms described in Case 1 strongly suggested acute coronary syndrome. It is well established that there are sex disparities in the incidence [21], presentation [22], diagnostics [23], and treatment of acute coronary syndrome. Possibly, this knowledge influenced the physicians' clinical judgement. Thus, increasing the probability that a male, compared to a female patient, with signs of both acute coronary syndrome and sepsis will be treated for acute coronary syndrome rather than sepsis.

The symptoms described in Case 2 indicated opioid withdrawal. Opioid addiction is less common in females compared to men [18], and men have a higher rate of receiving treatment for opioid withdrawal [24]. This may have swayed the physicians to suspect and focus treatment on opioid withdrawal in the case with the male patient compared to the case with the female patient. Thus, making sepsis a less likely diagnosis for men. However, not studied in the present investigation, one could speculate that the result could have been opposite if one of the constructed cases would have resembled medical conditions more common in the female sex, e.g., intoxication by oral sedatives.

Although both Cases 1 and 2 described patients with sepsis, the differential diagnoses of acute coronary syndrome and opioid withdrawal, respectively, were more often proposed than sepsis by the physicians included in the study. A minority of physicians made the correct sepsis diagnosis. The participating physician was most often not specifically trained in emergency medicine, infectious disease medicine, or intensive care medicine. This probably contributed to the poor rate of correct sepsis diagnosis and subsequent initial treatment. This study was not designed to study the effect of knowledge in sepsis treatment and hence, no such analyses were performed. However, the doctors working at the emergency room in the Swedish healthcare system are often inexperienced doctors from different specialties, seldom specialized in emergency care. This increases the applicability of this study in the Swedish setting. If the cases would have been more obvious regarding the symptomatology of sepsis or if the participating physicians would, to a higher degree, have been trained in sepsis management, the results could have been different. It might be the participating physicians' low rate of sepsis management training and the cases' resemblance to other diagnoses that paradoxically resulted in the higher rate of sepsis diagnoses in female patients. This could explain our diverging results compared to the clinical observations that males more often suffer sepsis compared to females [1] and that females are less likely to receive correct sepsis treatment [13, 14]. A small number of specialist physicians in clinical psychiatry were included in the study. The rationale for including psychiatrists was that in Case 2, the presentation resembled opioid withdrawal. One possible proposed initial measure could have been referral to psychiatry. The small amount of psychiatrists included did not influence the result (data not shown).

The study has several limitations. First, a questionnaire-based study can obviously never be totally representative of how the physicians would have reasoned in actual patients. Second, the anonymous design of the study made it impossible to approach the physicians who did not respond, thus leading to a fairly high share of nonresponders. Third, the study is a fairly small single-centre study which makes it hard to draw strong conclusions.

The design of the study was an innovative approach to be able to study the effect of gender. The clinical implication of the study is that clinical physicians should be aware that clinical decision-making can be influenced by gender, especially when assessing patients with sepsis and clinical conditions resembling septic symptoms. Gender medicine in conjunction with knowledge of septic symptoms and sepsis management should be an essential part in various stages of training for physicians, as well as in the continuing learning process after achieved medical specialty certification.

It would be of interest to conduct similar studies in other healthcare systems than the system currently studied and also to study whether the effect could be replicated in a population of physicians with a higher level of training in emergency medicine and sepsis management. Further, it would be of interest to study whether the observed effects of gender also exist in other diagnoses than sepsis.

## 5. Conclusion

In this questionnaire-based case study, a low proportion of Swedish physicians identified sepsis as an important differential diagnosis in four constructed cases. Female sex in patients rendered a higher probability for correctly identifying sepsis as an important differential diagnosis and proposing adequate initial treatment for sepsis. The study design is well suited for studying the way differential diagnoses are formed.

## Figures and Tables

**Table 1:** The distribution of the participants answering the two versions of the questionnaire with regard to sex, age, seniority, and medical specialty.

Version	A	B	*p*
*n* (%)	62	58	
Male sex *n* (%)	34 (55)	31 (51)	0.66
Responder age span *n* (%)			
20–30	14 (23)	13 (22)	0.89
30–40	21 (34)	24 (41)	0.43
40–50	12 (19)	12 (21)	0.78
50–60	10 (16)	5 (9)	0.25
60+	1 (2)	1 (2)	1.00
Responder position *n* (%)			
Intern	18 (29)	13 (22)	0.38
Resident	20 (32)	26 (45)	0.14
Consultant/senior consultant	24 (39)	17 (29)	0.25
Medical specialty *n* (%)			
Internal medicine specialties	19 (31)	18 (31)	0.91
Surgical specialties	10 (16)	10 (17)	0.88
Infectious diseases	3 (5)	4 (7)	0.64
Anaesthesia and intensive care	11 (18)	10 (17)	0.89
Psychiatry	2 (3)	2 (3)	1.00

Data are presented as *n* (%). The *p* values are results from Chi^2^ tests and Fisher's exact tests, where appropriate.

**Table 2:** Distribution of the participants' answers to the two versions of the questionnaire.

Version	Case 1			Case 2			Case 3			Case 4		
	A	B	*p*	A	B	*p*	A	B	*p*	A	B	*p*
	(male)	(female)		(female)	(male)		(female)	(female)		(male)	(male)	
*n*	62	58		62	58		62	58		62	58	
Correct identification and initial sepsis treatment	2 (4)	12 (21)	<0.01	25 (40)	13 (22)	<0.05	22 (35)	25 (43)	0.40	20 (32)	20 (34)	0.79
Sepsis among differential diagnoses	16 (26)	20 (34)	0.30	52 (84)	54 (93)	0.10	51 (82)	51 (88)	0.36	42 (68)	44 (76)	0.33
Proposed fluid among initial treatments	15 (24)	24 (41)	<0.05	35 (56)	21 (36)	<0.05	31 (50)	37 (64)	0.12	45 (73)	41 (71)	0.81
Proposed antibiotics among initial treatments	5 (8)	16 (28)	<0.05	41 (66)	34 (59)	0.33	35 (56)	33 (57)	0.91	32 (52)	29 (50)	0.83
Proposed ICU as the appropriate level of care	37 (60)	34 (58)	0.97	35 (56)	27 (47)	0.51	50 (80)	42 (72)	0.25	34 (55)	37 (64)	0.16

Data are presented as *n* (%). The *p* values are results from Chi^2^ tests and Fisher's exact tests, where appropriate.

## Data Availability

Data are available upon request to the corresponding author.
